# Lysine acetylation in cyanobacteria: emerging mechanisms and functions

**DOI:** 10.1042/BST20241037

**Published:** 2025-02-12

**Authors:** Xin Liu, Mingkun Yang, Feng Ge, Jindong Zhao

**Affiliations:** 1 School of Animal Science and Nutritional Engineering, Wuhan Polytechnic University, Wuhan, 430070, China; 2 State Key Laboratory of Freshwater Ecology and Biotechnology, Institute of Hydrobiology, Chinese Academy of Sciences, Wuhan 430072, China; 3 State Key Laboratory of Protein and Plant Genetic Engineering, College of Life Sciences, Peking University, Beijing 100871, China

**Keywords:** cyanobacteria, lysine acetylation, lysine acetyltransferase (KAT), lysine deacetylase (KDAC), metabolic pathways, photosynthesis, stress responses

## Abstract

Cyanobacteria are ancient and abundant photosynthetic prokaryotes that play crucial roles in global carbon and nitrogen cycles. They exist in a variety of environments and have been used extensively as model organisms for studies of photosynthesis and environmental adaptation. Lysine acetylation (Kac), a widespread and evolutionarily conserved protein posttranslational modification, is reversibly catalyzed by lysine acetyltransferases (KAT) and lysine deacetylases (KDACs). Over the past decade, a growing number of acetylated proteins have been identified in cyanobacteria, and Kac is increasingly recognized as having essential roles in many cellular processes, such as photosynthesis, energy metabolism, and stress responses. Recently, cGNAT2 and CddA were identified as KAT and KDAC in the model cyanobacterium *Synechococcus* sp. PCC 7002, respectively. The identified Kac regulatory enzymes provide novel insight into the mechanisms that globally regulate photosynthesis in cyanobacteria and potentially other photosynthetic organisms. This review summarizes recent progress in our understanding of the functions and mechanisms of lysine acetylation in Cyanobacteria. The challenges and future perspectives in this field are also discussed.

## Introduction

Cyanobacteria, the oldest oxygen photosynthetic prokaryotes, are responsible for a significant portion of global primary production [1,2]. They have also played a crucial role in the evolution of chloroplasts in plants and algae through endosymbiosis [3]. These photosynthetic microorganisms exhibit remarkable adaptability, enabling them to thrive in a diverse range of environments, from oceans and freshwater bodies to extreme habitats such as deserts, icebergs, polar regions, and hot springs [2]. This extensive adaptability is largely attributed to their ability to evolve strategies that allow them to survive in diverse environments, including the utilization of reversible post-translational modifications (PTMs) as a rapid and efficient way to respond to changing environmental conditions [4].

PTMs encompass the covalent attachment of diverse functional groups, including phosphates, acetates, small proteins, lipids, and carbohydrates, to amino acids subsequent to protein synthesis [5]. These modifications fundamentally alter the chemical properties and structures of amino acids, thereby influencing protein function. To date, research has identified over 600 different types of PTM in various proteins, with phosphorylation, acetylation, and ubiquitination among the most extensively studied [6]. In particular, lysine acetylation is a prevalent PTM that involves the addition of an acetyl group to the ε-amino group of lysine residues [7,8]. Lysine acetylation neutralizes the positive charges of lysine, thus endowing the modified protein with new properties and affecting its functionality [7,8].

Lysine acetylation was first discovered in histones in 1964, marking the inception of a burgeoning field of study [9]. Over the next three decades, it became clear that this modification exerts a profound influence on histone function, shaping chromosomal architecture and orchestrating gene expression [10–12]. Advances in proteomic methodologies, including mass spectrometry (MS) and antibody affinity purification, have transformed our understanding of acetylation, unveiling a plethora of lysine acetylation sites in both the prokaryotic and eukaryotic kingdoms [13,14].

The first study of lysine acetylation in cyanobacteria was reported in 2008, where Gali et al. identified lysine-acetylated AbrB-like protein in *Aphanizomenon ovalisporum*, thus highlighting a possible regulatory impact of acetylation in cyanobacterial gene expression [15]. Subsequent studies by Guskov et al. and Baniulis et al. identified acetylated proteins within the cyanobacterial photosynthetic system, including ferredoxin Pet, the cytochrome protein PsbF, and the photosystem II protein PsbJ, pointing to their potential role in the regulation of photosynthesis [16,17]. Building on these early discoveries, recent advances in proteomics have identified numerous lysine-acetylated proteins in various cyanobacterial species, thus constructing regulatory networks of lysine acetylation [18–21]. Functional studies have also highlighted the critical regulatory role of lysine acetylation in the regulation of key processes in cyanobacteria, particularly photosynthesis and carbon metabolism [18–21]. Here, we recapitulate the recent progression of lysine acetylation research in cyanobacteria, focusing on its role in the regulation of photosynthesis, metabolism, and stress responses. These insights offer vital theoretical foundations for understanding cyanobacterial growth and its adaptation to environmental changes.

## Functions of lysine acetylation in cyanobacteria

Advancements in proteomics, instrumentation, and bioinformatics have significantly advanced the research on PTMs in cyanobacteria. Comprehensive investigations have mapped the landscape of lysine acetylation, identifying numerous acetylated lysine residues in various cyanobacterial species [18–21]. To better understand the diverse functions of lysine acetylation in these organisms, we compiled data from proteomic studies on lysine acetylation in cyanobacteria, including *Synechocystis* sp. PCC 6803 [18], *Synechococcus* PCC 7002 [19,21], and *Nostoc flagelliforme* [20]. Lysine acetylation in cyanobacteria is prevalent in a wide array of proteins involved in an assortment of biological processes, such as metabolism, photosynthesis, carbon metabolism, the two-component system (TCS), and reactive oxygen species (ROS) signaling.

### Photosynthesis

**Figure 1:**
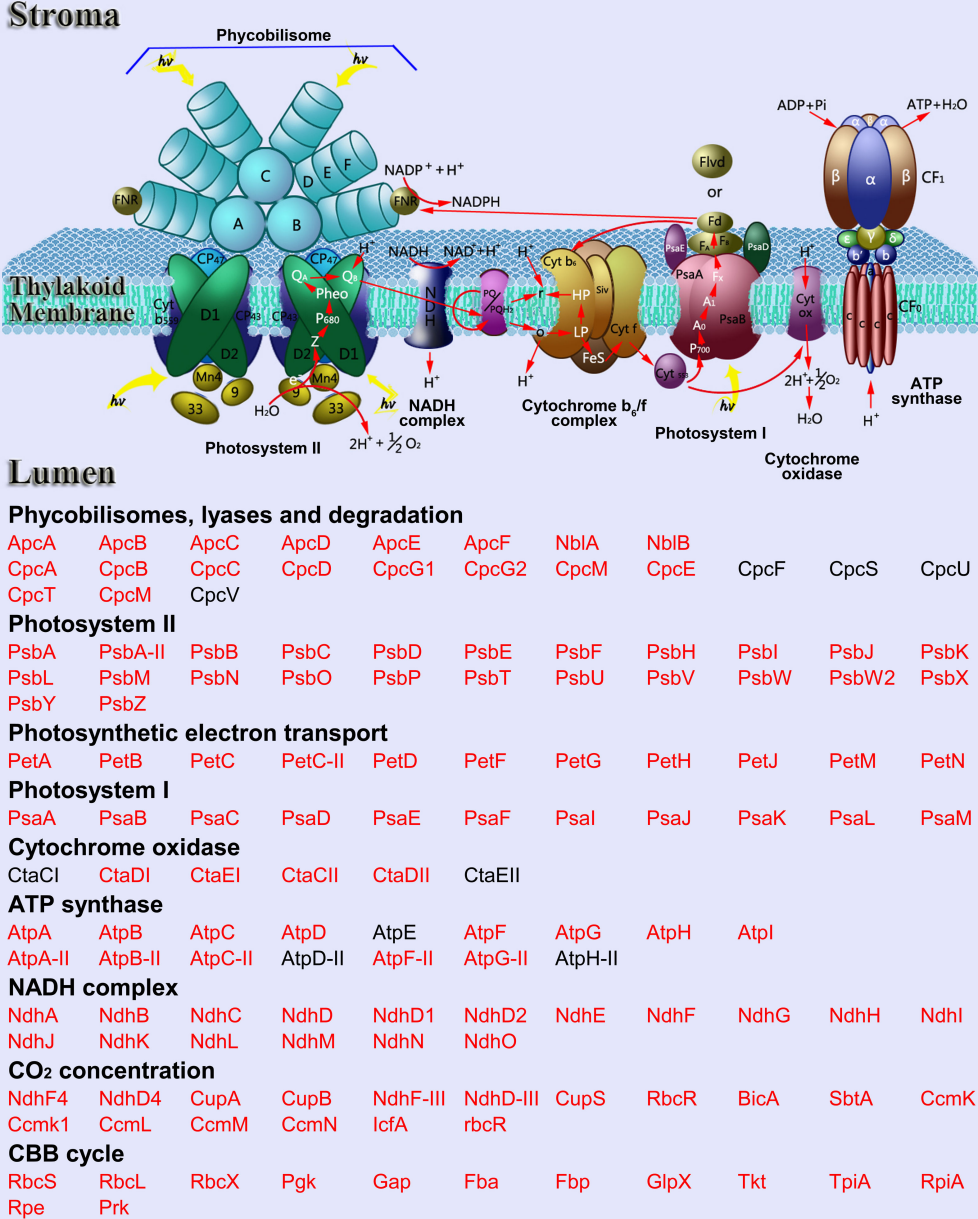
Lysine acetylation modified proteins in photosynthesis in cyanobacteria. The identified acetylated proteins were highlighted in red.

Photosynthesis, the process by which photoautotrophic organisms convert light energy into ATP and NADPH, is crucial for cyanobacterial survival [22,23]. Recent studies have highlighted the significance of cyanobacterial lysine-acetylated proteins in photosynthesis [19,21]. As shown in Figure 1, a significant portion of these proteins are involved in the photosynthetic pathway, impacting both light-dependent reactions and the Calvin cycle. Almost all major photosynthesis-related complexes harbor acetylated proteins, including photosystem I, photosystem II, the NAD(P)H dehydrogenase (NDH)-1 complex, allophycocyanin, phycocyanin, cytochrome b_6_/f complex, and ATP synthase complex (Figure 1).

Lysine acetylation plays a critical regulatory role in cyanobacterial photosynthesis. For example, the PsbO protein, a key component of the oxygen-evolving complex (OEC) in photosynthesis, facilitates the transition between the five oxidation states (S_1_, S_2_, S_3_, S_4_, and S_0_) of the OEC, with the S_4_ state crucial for water splitting and oxygen evolution [24–26]. The research by Chen et al. identified 36 acetylated proteins involved in photosynthesis, demonstrating that acetylation of PsbO in lysine 190 significantly influences the stability of the S_2_ and S_3_ states, thus altering the transition of the S state from S_3_ to S_4_ and subsequently to S_0 (19)_. This acetylation serves as a regulatory mechanism, modulating the efficiency of the OEC and the overall photosynthetic performance [19]. However, despite these functional insights, the precise stoichiometry or site occupancy of acetylation in PsbO lysine 190 remains unknown. Although combining immunoenriched proteomics and functional studies is valuable for measuring relative acetylation fold changes, they do not provide direct information on stoichiometry or occupancy at individual sites. The stoichiometry of lysine acetylation is crucial in interpreting its biological significance. Recently, Jia et al. discovered 38 acetylated proteins associated with photosynthesis, focusing on the acetylated proteins within the NDH-1 complex [21]. This complex is essential for respiration, cyclic electron flow around photosystem I, and CO_2_ uptake in cyanobacteria [27–29]. Acetylation of lysine 89 in NdhJ, a hydrophilic subunit of the NDH-1 complex, influences the enzymatic activity of NdhJ, thus affecting photosynthetic electron flow and cyanobacterial growth [21]. Similar regulatory mechanisms are also observed in the dark reactions of photosynthesis. For example, acetylation levels of the large subunit of ribulose-1,5-bisphosphate carboxylase/oxygenase (RbcL), a key enzyme in carbon fixation, are negatively correlated with its activity [21]. These findings underscore the role of lysine acetylation in modulating the activities of key enzymes and proteins in both light-dependent reactions and Calvin cycle phases of photosynthesis.

### Carbon metabolism/energy metabolism

**Figure 2:**
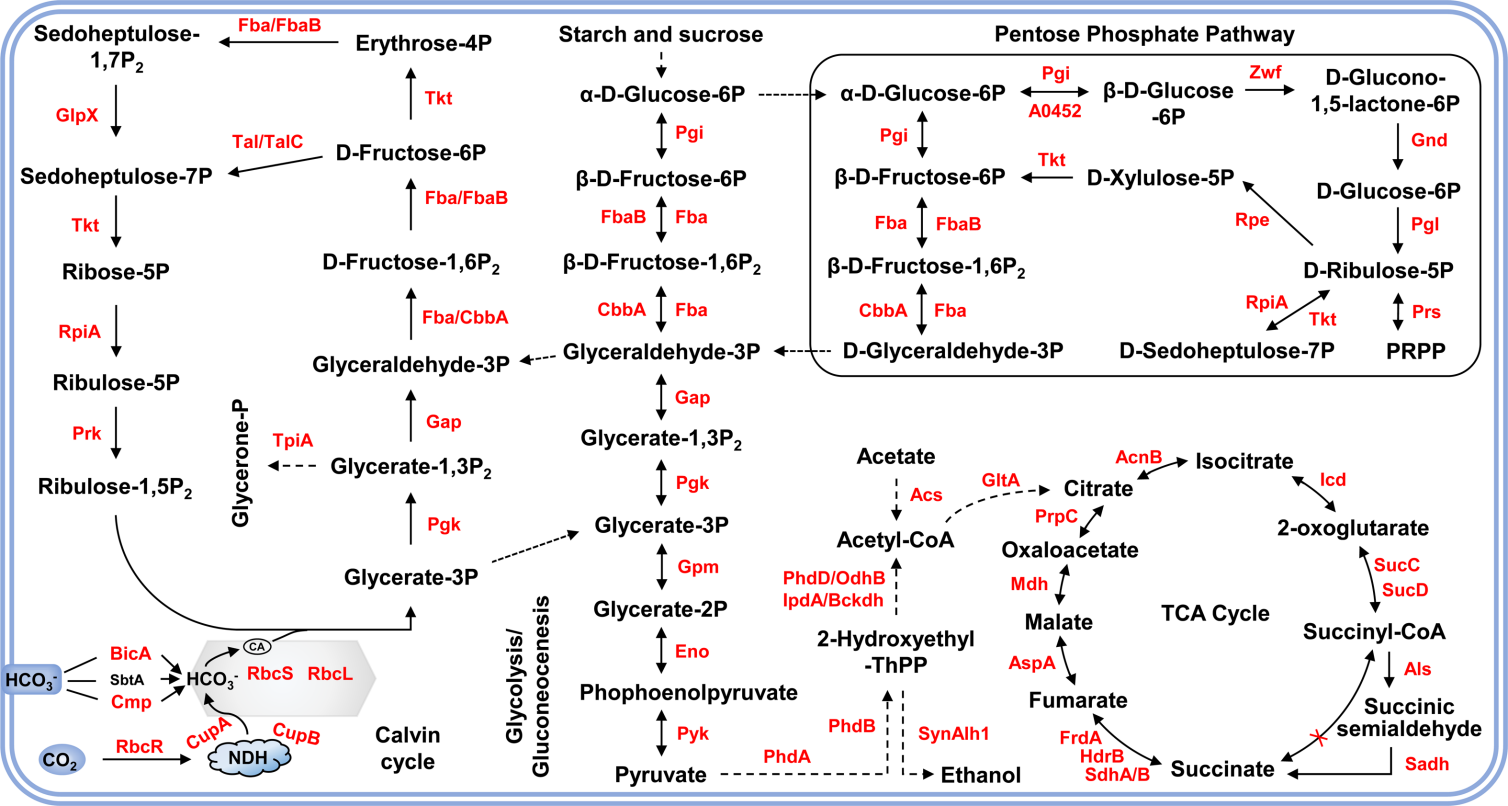
Lysine acetylation modifications in metabolism in cyanobacteria. The identified acetylated proteins were highlighted in red.

Proteomic analyses reveal that lysine acetylation in cyanobacteria extends to a variety of proteins involved in ribosome function and various metabolic pathways, including the tricarboxylic acid cycle, starch and sucrose metabolism, and the pentose phosphate pathway (Figure 2). In particular, almost all key enzymes in these pathways harbor acetylation sites, highlighting the influence of lysine acetylation on metabolic regulation. Among these enzymes, two enzymes, fructose-1,6-bisphosphatase (F/SBPase) and glyceraldehyde-3-phosphate dehydrogenase (GAPDH), are particularly important for cyanobacterial metabolism. F/SBPase is essential for gluconeogenesis and the Calvin cycle, catalyzing fructose-1,6-bisphosphate hydrolysis of fructose-1,6-bisphosphate [30], whereas GAPDH plays a central role in glycolysis and gluconeogenesis, facilitating energy production and carbohydrate metabolism through redox reactions [31,32]. In *Synechococcus* PCC 7002, lysine acetylation significantly affects the enzymatic activities of F/SBPase and GAPDH. Acetylation at lysine 184, located at the catalytic site of F/SBPase, modulates the activity of this enzyme [33]. Similarly, acetylation of lysine 272 at the catalytic site of GAPDH activates the activity of this enzyme. These observations highlight the role of lysine acetylation as a critical regulatory mechanism for maintaining energy balance within cyanobacterial cells [21]. This dynamic regulation of enzyme activity through acetylation enables cyanobacteria to quickly adjust to changing metabolic demands and environmental conditions.

### Stress responses and other biological processes

Cyanobacteria have developed intricate molecular mechanisms to cope with environmental stresses, with lysine acetylation emerging as a particularly efficient and cost-effective regulatory strategy [18,20]. A recent study by Wang et al. underscores the crucial role of lysine acetylation in fine-tuning *N. flagelliforme* responses to dehydration stress [20]. Specifically, lysine acetylation levels of proteins related to photosynthesis, particularly RbcL, were found to change in response to dehydration [20]. As a key enzyme in carbon fixation, the reduced lysine acetylation of RbcL suggests that this modification may act as a negative regulator of its activity under stress conditions [20]. Similar patterns in acetylation levels were also observed in other Calvin cycle enzymes, indicating that lysine acetylation serves as a fine-tuning mechanism for photosynthesis during dehydration [20].

**Figure 3:**
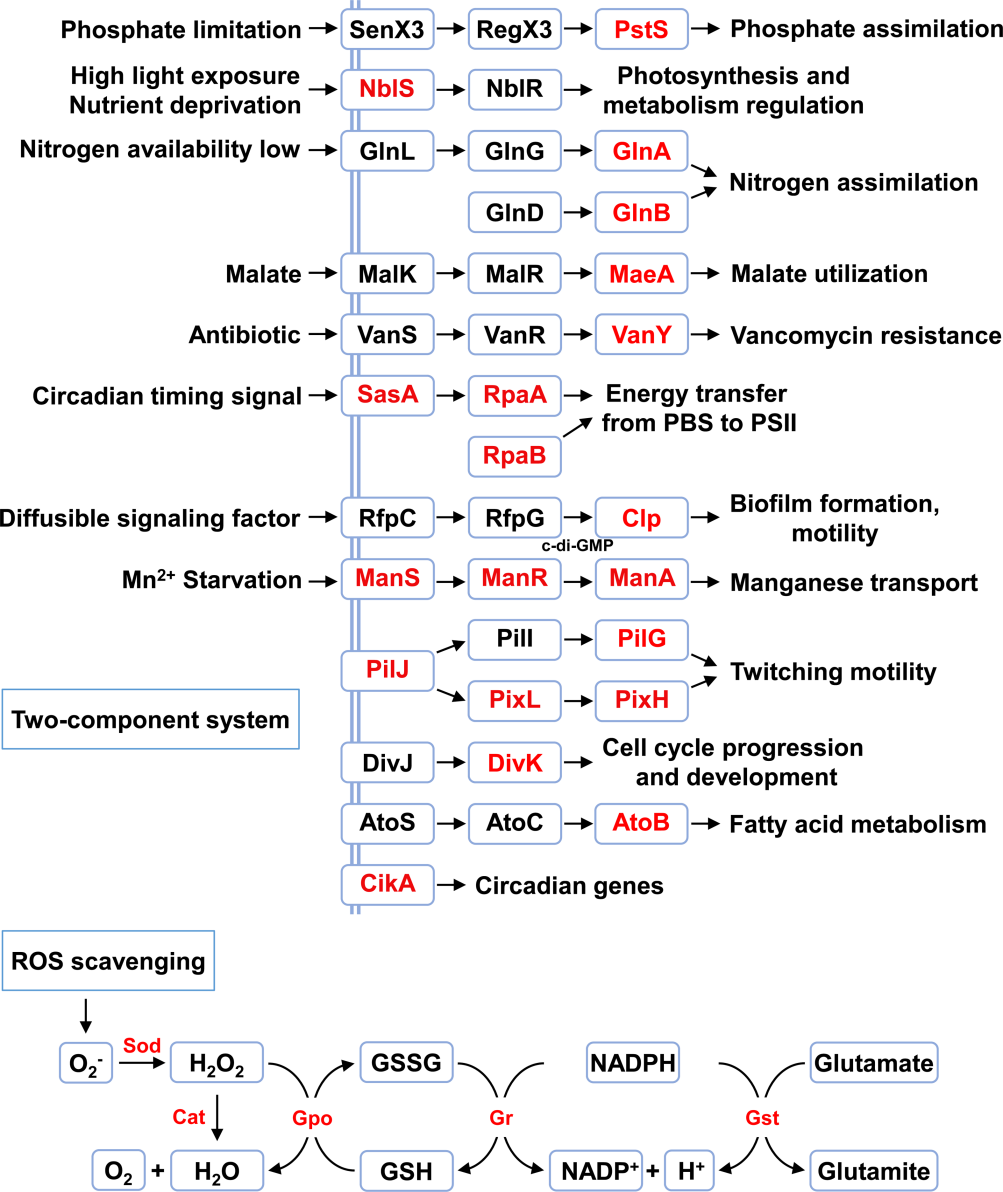
Lysine acetylation modifications in stress responses in cyanobacteria. The identified acetylated proteins were highlighted in red.

Beyond lysine acetylation, cyanobacteria also rely on TCSs as an important regulatory mechanism to sense environmental changes and adjust their responses [34–37]. These systems, which consist of sensor histidine kinases and response regulators, enable cyanobacteria to quickly adapt to fluctuating conditions [38,39]. In particular, several TCS proteins, such as signal transduction histidine kinase (NblS), adaptive response sensory kinase (SasA), and two-component response regulator (PilG), are subject to acetylation (Figure 3). Specifically, NblS is a membrane-associated sensor histidine kinase with a PAS domain, which transmits intracellular signals through the phosphorylation of response regulators [40]. These signals are predominantly associated with environmental stress responses, such as high exposure to light and nutrient deprivation [40–43]. The lysine acetylation of NblS may influence its kinase activity, thus contributing to the regulation of these environmental response processes. This interaction with lysine acetylation underscores a collaborative network of regulatory mechanisms that enhances cyanobacteria’s ability to cope with environmental changes.

Furthermore, cyanobacteria frequently encounter oxidative stress due to environmental fluctuations, which requires an efficient ROS scavenging system to alleviate oxidative damage [44–48]. As shown in Figure 3, many key enzymes in this system, including superoxide dismutase (SOD), catalase (CAT), glutathione peroxidase (GPX), glutathione S-transferase (GST), and glutathione reductase (GR), are acetylated, suggesting a possible regulatory role for lysine acetylation in the response to oxidative stress. Research on *N. flagelliforme* has shown significant changes in lysine acetylation of these antioxidant enzymes under dehydration stress [20]. These findings highlight the regulatory function of acetylation in the ROS scavenging system, helping cyanobacteria maintain redox balance and survive in adverse conditions.

In addition, N-terminal acetylation plays a unique regulatory role in the production of various peptide toxins in cyanobacteria. Acetylation of AbrB-like protein (antibiotic resistance protein B) has been implicated in the regulation of cylindrospermopsin production and the adaptive response of *Aphanizomenon* to changing environmental conditions [15]. In *Nostoc sp*. 152, the O-acetylated microcystin variants exhibit toxicity levels comparable to the most toxic microcystin variants, suggesting a critical role for acetylation in regulating toxin production in cyanobacteria [49]. In *Planktothrix agardhii*, acetylation has been shown to be essential for converting the linear precursor prepeptide into mature tricyclic microviridin K [50]. Collectively, these findings highlight the critical role of acetylation in modulating the production and toxicity of peptide-based secondary metabolites in cyanobacteria, enabling these organisms to adapt to environmental pressures and maintain ecological competitiveness.

## Enzymes involved in lysine acetylation and deacetylation

**Figure 4:**
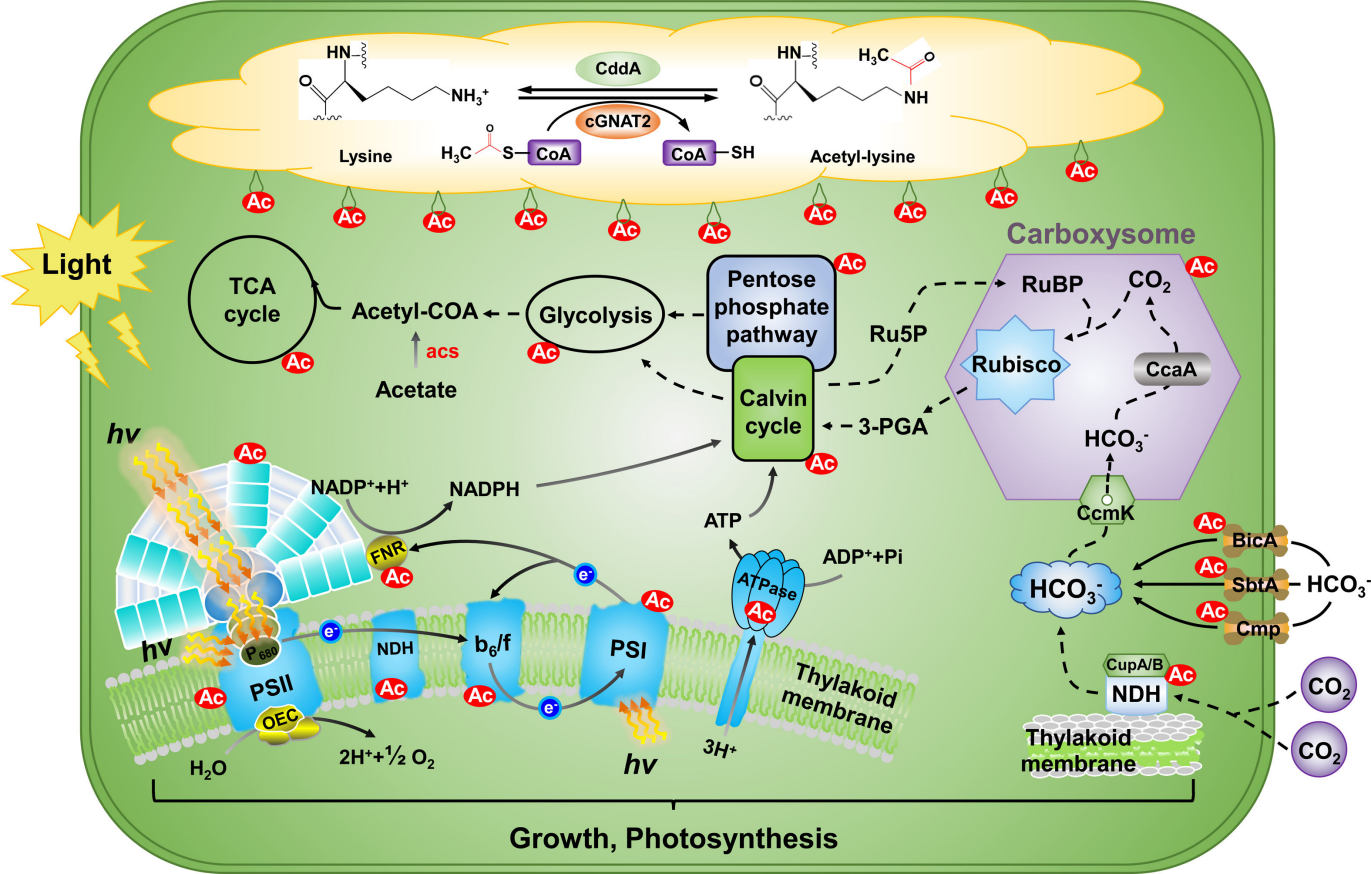
Functions and mechanisms of cGNAT2 and CddA in cyanobacteria.

Lysine acetylation is a dynamic and reversible PTM that is enzymatically catalyzed by KAT and KDACs (Figure 4) [7]. Additionally, nonenzymatic acetylation can occur through reactive acetyl derivatives, including acetyl phosphate (AcP) and acetyl adenylate (Ac-AMP) [51,52]. This study compiles information on acetylated proteins and their specific sites that are regulated by the cyanobacterial acetyltransferase cGNAT2 [21] and the deacetylases CddA [33]. Using these data, we constructed functional networks that map out lysine-acetylated proteins and their associations with both cGNAT2 and CddA (Figure 4). These two enzymes may contribute to the dynamic balance between acetylation and deacetylation, further fine-tuning important pathways such as metabolism and photosynthesis in *Synechococcus* sp. PCC 7002.

### KATs in cyanobacteria

KATs regulate protein function by transferring an acetyl group to the ε-amino group of a targeted lysine residue. In eukaryotes, KATs are classified into three families: GNAT (Gcn5-related N-acetyltransferase), MYST (MOZ, Ybf2/Sas3, Sas2, and Tip60), and p300/CBP (CREB-binding protein) [53,54]. The GNAT superfamily is the most evolutionarily conserved, found in bacteria, eukaryotes, and archaea, while the p300/CBP and MYST families are found only in eukaryotes [55–57]. KATs use a general acid/base mechanism, where key residues such as glutamate or aspartate deprotonate the amine group of the substrate, facilitating the transfer of an acetyl group from acetyl-CoA (AcCoA) to the target protein [57–60].

In prokaryotes, the known prokaryotic KATs so far belong to the GNAT family and YopJ effector family [53]. The first prokaryotic KAT, YifQ (also known as Pat, GNAT family), was identified in *Salmonella enterica*, where it regulates the activity of acetyl-CoA synthetase (Acs) and plays a key role in bacterial metabolism [52,53]. Subsequent investigations revealed that YifQ homologues play important roles in the regulation of enzyme activity, protein stability, stress response, and cellular metabolism in various bacterial species [53]. In 2018, Christen et al. conducted a systematic screening of *Escherichia coli* using an acetylation-defective strain of *E. coli* that lacked the donor of acetylphosphate (AcP), KAT (YfiQ), acetyl-CoA synthase (Acs), and deacetylase (CobB). They identified four additional enzymes with KAT activity: RimI, YiaC, YjaB, and PhnO, all of which are members of the GNAT family [61]. In particular, YopJ effectors are only found in prokaryotes, which depend on a novel acetyltransferase domain to acetylate-speciﬁc residues of substrate proteins, further inhibiting enzymatic activation [53,62].

In cyanobacteria, bioinformatic analysis has predicted several potential KATs, but recent research confirmed the acetyltransferase activity in *Synechococcus* sp. PCC 7002, termed cyanobacterial Gcn5-related N-acetyltransferase (cGNAT2) [21]. Structural simulations revealed that cGNAT2 possesses the characteristic features of the GNAT superfamily, comprising seven β-strands and four α-helices, and forming a typical ‘P-loop’ structure that is critical for substrate binding and catalytic function [21,63,64]. Structural simulations and site-directed mutagenesis identified eight key amino acid residues (Val54, Trp56, Trp 105, Gln114, Gly119, Leu122, Met123, and Phe141) essential for the activity of cGNAT2. Functional studies suggest that cGNAT2 affects cyanobacterial growth and electron transport processes [21]. Furthermore, while the unique polyketide-like synthase SxtA from the cyanobacterium *Cylindrospermopsis raciborskii* contains a GNAT domain capable of substrate decarboxylation, its potential acetyltransferase activity remains to be elucidated [65]. Similarly, the polyketide synthase responsible for curacin A biosynthesis in the marine cyanobacterium *Lyngbya majuscule* contains a GNAT domain with bifunctional decarboxylase and S-acetyltransferase activities, underscoring the various functional roles of GNAT-containing enzymes in secondary cyanobacterial secondary metabolism [66].

### KDACs in cyanobacteria

KDACs, often referred to as histone deacetylases (HDACs) due to their role in histone modification, are responsible for the removal of acetyl groups from lysine residues [7]. These enzymes are classified into two categories: Zn^2+^-dependent HDACs and NAD^+^-dependent sirtuins. Zn^2+^-dependent HDACs act as hydrolases that release acetate, whereas sirtuins use NAD^+^ as a cosubstrate, producing nicotinamide and 2′-O-acetyl-ADP-ribose as byproducts [67–69]. These enzymes are highly conserved in both eukaryotic and prokaryotic organisms and play a pivotal role in genomic stability, transcriptional regulation, cellular metabolism, and responses to cellular stress responses [7,54,70–73].

The first prokaryotic sirtuin deacetylase, CobB, was identified in *Salmonella enterica*, where it deacetylates lysine 609 of acetyl-CoA synthetase, thus regulating enzymatic activity and cellular metabolism [74]. Following this discovery, Zn^2+^-dependent HDACs were also reported in prokaryotes [75–76]. For example, Crosby et al. described a Zn^2+^-dependent HDAC, LdaA, regulating the enzyme activity of branched-chain amino acid aminotransferase by decreasing its acetylation levels [75]. In 2015, Tu et al. identified a novel deacetylase, YcgC, in *E. coli*. Unlike conventional HDACs and sirtuins, YcgC uses Ser200 as the nucleophile during deacetylation and can catalyze the deacetylation of Lys52/62 in the transcriptional regulator RutR, thus regulating gene transcription [77].

Although cyanobacteria lack CobB and YcgC homologs, members of the HDAC family have been identified in this group [33]. However, their enzymatic activities were not characterized until recently. Liu et al. identified the first deacetylase in the cyanobacterium *Synechococcus* sp. PCC 7002, known as CddA, which exhibits both deacetylase and depropionylase activities [33]. The analysis of CddA crystal structure revealed a unique α/β fold and a binding site of Zn^2+^ essential for its deacylase activity. Its substrate-binding pocket is composed of amino acid residues His126, His127, Phe136, Tyr192, Leu255, and Tyr287. Further structural analysis and site-directed mutagenesis demonstrated that key residues (His126, His127, and Tyr287) play a crucial role in modulating the enzyme’s catalytic function and substrate recognition. Furthermore, CddA was shown to regulate photosynthesis and carbon metabolism in cyanobacteria [33]. Currently, deacetylases in cyanobacteria remain largely unexplored. Through genome annotation and sequence alignment (https://www.ncbi.nlm.nih.gov), we identified several proteins in cyanobacteria annotated as putative deacetylases. However, their precise biochemical functions and regulatory roles require further experimental validation.

## Conclusions

Lysine acetylation has emerged as a multifaceted regulatory mechanism in cyanobacteria, intricately involved in modulating various biological processes such as photosynthesis, metabolism, and stress responses. The identification and characterization of acetyltransferases and deacetylases, particularly cGNAT2 and CddA, have provided valuable information on the molecular mechanisms governing this PTM.

The landscape of acetylation within cyanobacteria is extensive and encompasses proteins essential for energy production, carbon fixation, and adaptation to environmental stressors. Proteomic studies have revealed regulatory networks where lysine acetylation exerts a wide influence on the physiology of cyanobacteria. For instance, the acetylation of photosynthetic proteins like PsbO, NdhJ, and RbcL fine-tunes photosynthetic processes. Similarly, acetylation of metabolic enzymes such as F/SBPase and GAPDH underscores the dynamic regulation of central metabolic pathways. In addition, lysine acetylation plays a critical role in stress response, as seen in its modulation of antioxidant enzymes and proteins involved in the ROS scavenging system, which is critical for the survival of cyanobacterials under oxidative stress. The interaction between lysine acetylation and TCSs further underscores the intricate regulatory networks that enable cyanobacteria to sense and adapt to changing environmental conditions.

Looking forward, several key challenges and opportunities await in the study of lysine acetylation in cyanobacteria. First, a more comprehensive understanding of the enzymatic activities and specific targets of acetyltransferases and deacetylases is required to elucidate the full scope of their regulatory roles. Notably, the functions of uncharacterized acetyltransferases (SYNPCC7002_A0096) and deacetylases (SYNPCC7002_A1994, SYNPCC7002_A1628) in cyanobacteria remain largely unexplored, and their potential regulatory targets could shed new light on cyanobacterial biology. Second, further research is needed on how lysine acetylation modulates protein function, particularly within photosynthetic and metabolic pathways. For instance, does lysine acetylation affect the stability of cyanobacterial photosynthetic complexes, thus influencing photosynthetic efficiency? Integrated computational methods could help predict acetylation sites and simulate the impact of acetylation on protein structure and function, with further validation through crystallography. Third, exploring the role of lysine acetylation in cyanobacterial adaptation to environmental stresses could reveal novel strategies to improve stress tolerance in other organisms, including crop species. Additionally, acetylation does not act in isolation; it often competes with other central metabolites for lysine residues. As we endeavor to truly understand the inner workings of the cell, understanding the interplay between acetylation and other PTMs presents a significant challenge, but it is crucial for a comprehensive understanding of cellular regulation. Lastly, potential applications for understanding lysine acetylation in cyanobacteria should not be overlooked. From biotechnological perspectives, harnessing the regulatory power of lysine acetylation could lead to the engineering of cyanobacteria with improved photosynthetic efficiency or enhanced stress tolerance. Such advances could contribute to bioenergy production, carbon capture, and the development of cyanobacteria-based environmental remediation strategies.

In summary, lysine acetylation serves as a key regulatory hub in cyanobacteria, with significant implications for its physiology and potential applications. Unraveling the complexity of this regulatory mechanism will not only deepen our understanding of cyanobacterial biology but will also open avenues for innovative biotechnological advancements.

**Perspectives**

- Lysine acetylation and its regulatory enzymes are increasingly recognized to play essential roles in various cellular processes in cyanobacteria.
- We have outlined the recent progress in our understanding of the functions and mechanisms of lysine acetylation and its regulatory enzymes in cyanobacteria.
- Further work is required to understand the enzymatic activities and specific targets of KATs and KDACs to elucidate the full scope of their regulatory roles in cyanobacteria.

## Open Access

This article has been published open access under our Subscribe to Open programme, made possible through the support of our subscribing institutions, learn more here: https://portlandpress.com/pages/open_access_options_and_prices#conditional

## Abbreviations

AcP: acetyl phosphate
CAT: catalase
GPX: glutathione peroxidase
GR: glutathione reductase
GST: glutathione S-transferase
HDACs: histone deacetylases
KAT: lysine acetyltransferases
KDACs: lysine deacetylases
MS: mass spectrometry
OEC: oxygen-evolving complex
PTM: post-translational modifications
ROS: reactive oxygen species
SOD: superoxide dismutase
TCA: tricarboxylic acid
TCSs: two-component systems
